# Supplementary material for: Vitamin C and E Treatment Blunts Sprint Interval Training–Induced Changes in Inflammatory Mediator-, Calcium-, and Mitochondria-Related Signaling in Recreationally Active Elderly Humans
Source: Antioxidants (Basel). 2020 Sep 17;9(9):879. doi: 10.3390/antiox9090879 (PMC7555371; doi:10.3390/antiox9090879)
Supplement: Supplementary file 1 [file antioxidants-09-00879-s001.pdf]

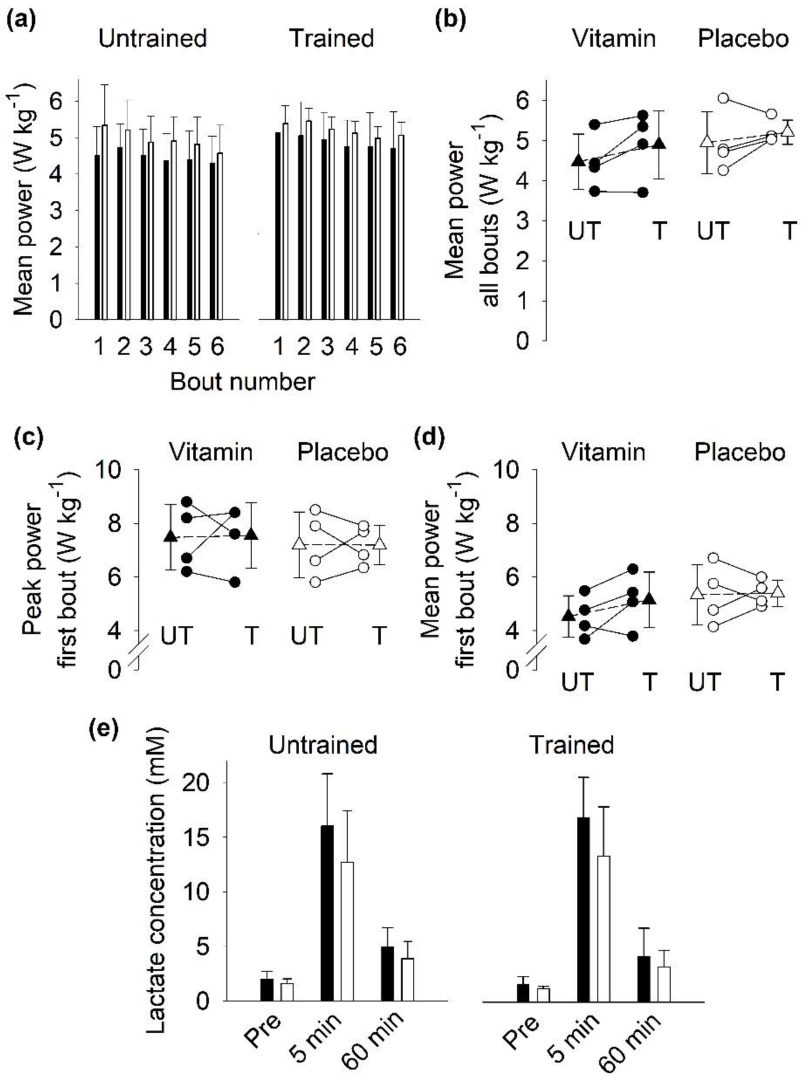

Figure S1. The all-out cycling performance was not notably affected by three weeks of SIT in either the vitamin or the placebo group. (a) Mean power output during each of the six bouts of 30 s all-out cycling in the untrained (first SIT session) and trained (last SIT session) states of participants receiving vitamins (n = 4) or placebo (n = 4). (b) Mean power during all six cycling bouts in the untrained (U) and trained (T) states. Peak power (c) and mean power (d) during the first cycling bout in the untrained and trained states. Data from each individual (●,○) and mean data (▲,Δ); n = 4 in each group. Two-way repeated measures ANOVA revealed no training effect or difference between the vitamin and the placebo groups for any of the measured parameters. (e) Mean blood lactate before (Pre) and 5 and 60 min SIT sessions performed in the untrained and trained states by subjects receiving vitamins (n = 5) or placebo (untrained state, n = 5; trained state, n = 3). Data are presented as mean ± SD; vitamin group (black bars and symbols); placebo group (white bars and symbols). Two-way repeated measures ANOVA showed no difference between the vitamin and the placebo groups at any time point.

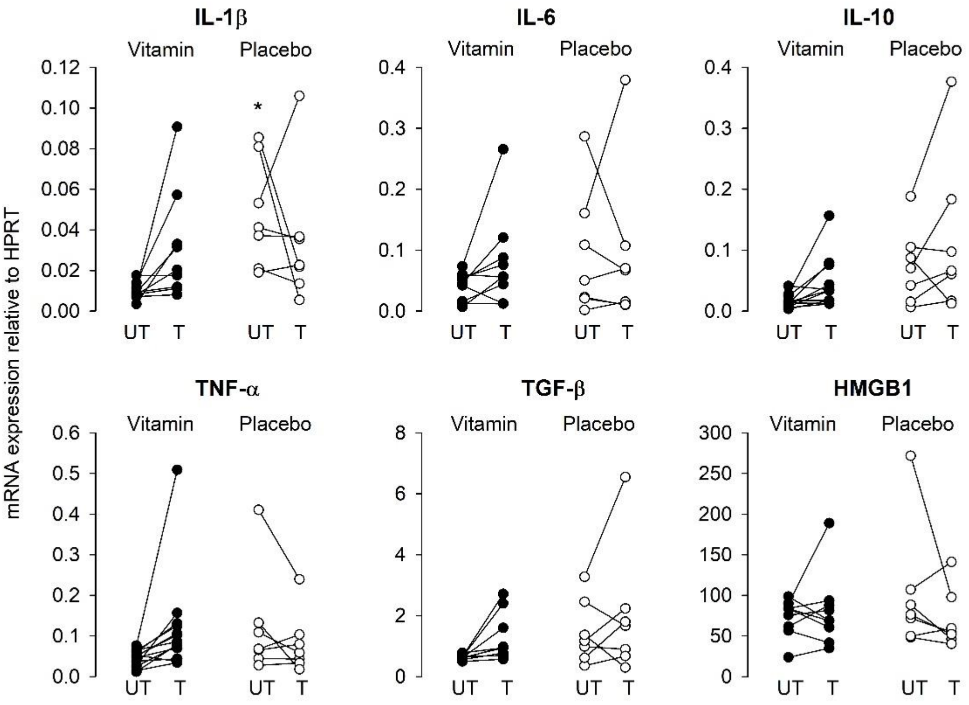

Figure S2. Gene expression of inflammatory mediators in the untrained state (UT, i.e. before the first SIT session) and the trained state (T; i.e. before the last SIT session). Data from vitamin-treated (●) and placebo-treated (○) individuals. mRNA expressions presented as $2^{-\Delta C_t}$, where $\Delta C_t = C_t[\text{Target}] - C_t[\text{Housekeeping}]$. Two-way repeated measures ANOVA for each revealed a significantly higher (*, $P < 0.05$) mRNA expression of IL-1β in the placebo than in the vitamin group; no other differences were detected between the two groups or between the untrained and trained states within each group.

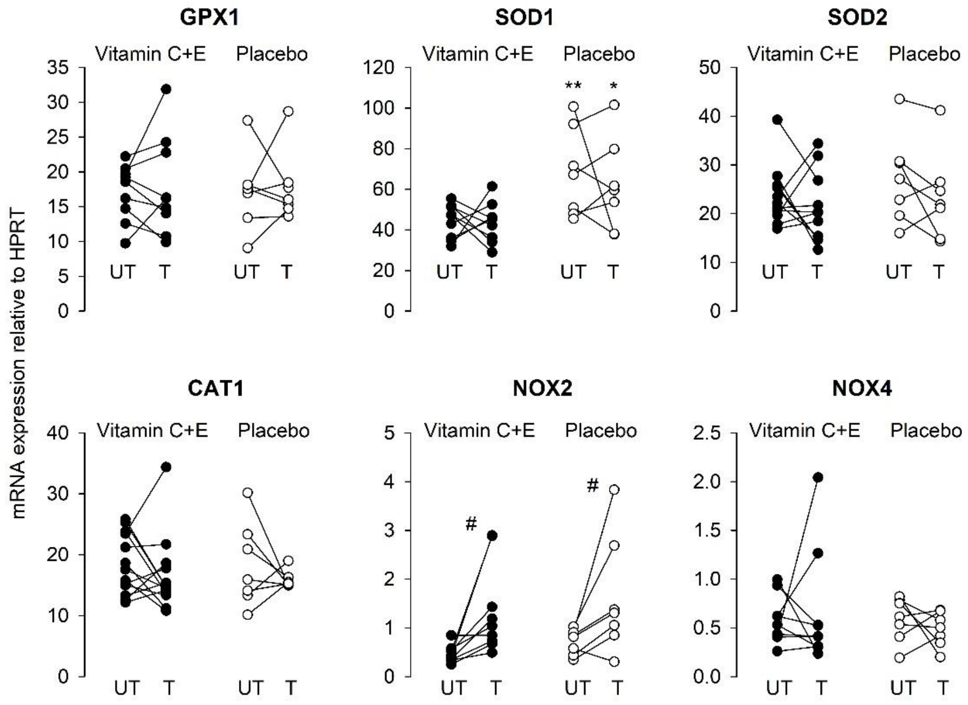

Figure S3. Gene expression of ROS-related proteins in the untrained state (UT, i.e. before the first SIT session) and the trained state (T; i.e. before the last SIT session). Data from vitamin-treated (●) and placebo-treated (○) individuals. mRNA expressions presented as $2^{-\Delta Ct}$, where $\Delta C_t = C_t$[Target] - C_t[Housekeeping]. Two-way repeated measures ANOVA for each gene revealed a significantly training effect of NOX2 both in the vitamin and placebo groups (#, $P < 0.05$) and higher mRNA expression of SOD1 in the placebo than in the vitamin group both in untrained (**, $P < 0.01$) and trained (*, $P < 0.05$) states; no other differences were detected between the two groups or between the untrained and trained states within each group.

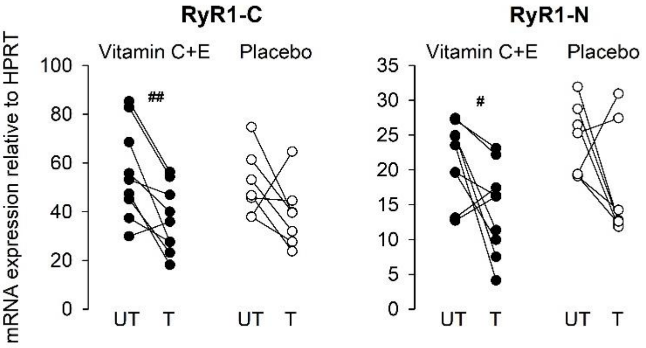

Figure S4. Gene expression of RyR1-C and -N in the untrained state (UT, i.e. before the first SIT session) and the trained state (T; i.e. before the last SIT session). Data from vitamin-treated (●) and placebo-treated (○) individuals. mRNA expressions presented as $2^{-\Delta C_t}$, where $\Delta C_t = C_t[\text{Target}] - C_t[\text{Housekeeping}]$. Two-way repeated measures ANOVA for each gene revealed a significantly negative training effect of both RyR1-C and -N both in the vitamin group (#, $P < 0.05$; ## $P < 0.01$); no other differences were detected between the two groups or between the untrained and trained states within each group.

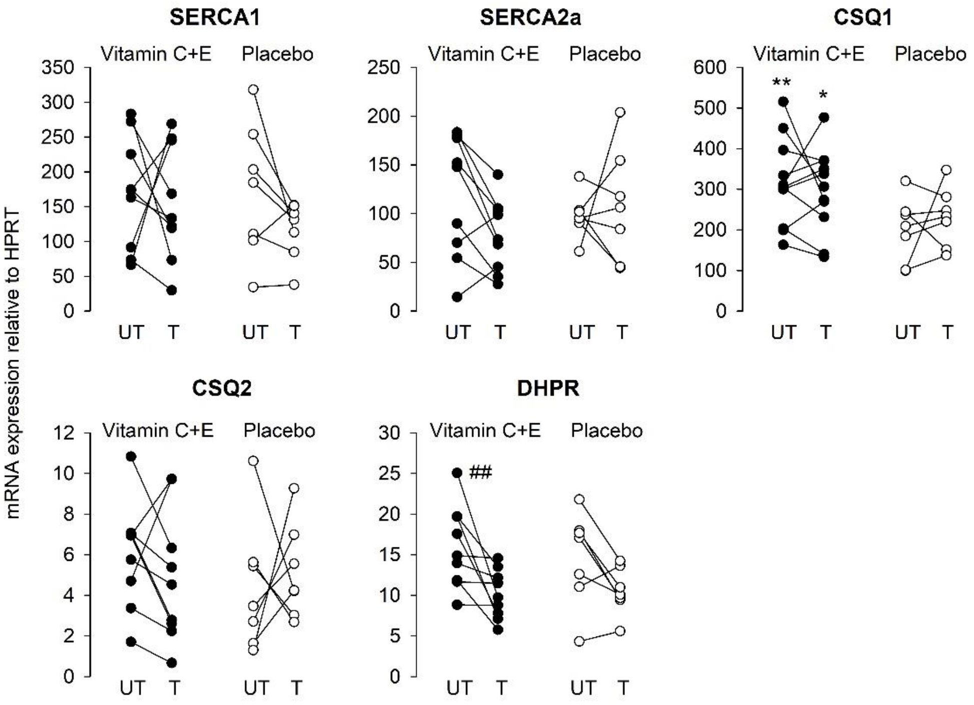

Figure S5. Gene expression of SR Ca^{2+}-handling proteins (other than RyR1) in the untrained state (UT, i.e. before the first SIT session) and the trained state (T; i.e. before the last SIT session). Data from vitamin-treated (●) and placebo-treated (○) individuals. mRNA expressions presented as $2^{-\Delta C_t}$, where ΔC_t = C_t[Target] - C_t[Housekeeping]. Two-way repeated measures ANOVA for each gene revealed a significantly negative training effect of DHPR in the vitamin group (## $P < 0.01$) and higher mRNA expression of CSQ1 in the vitamin than in the placebo group both in the untrained (**, $P < 0.01$) and trained (*, $P < 0.05$) states; no other differences were detected between the two groups or between the untrained and trained states within each group.

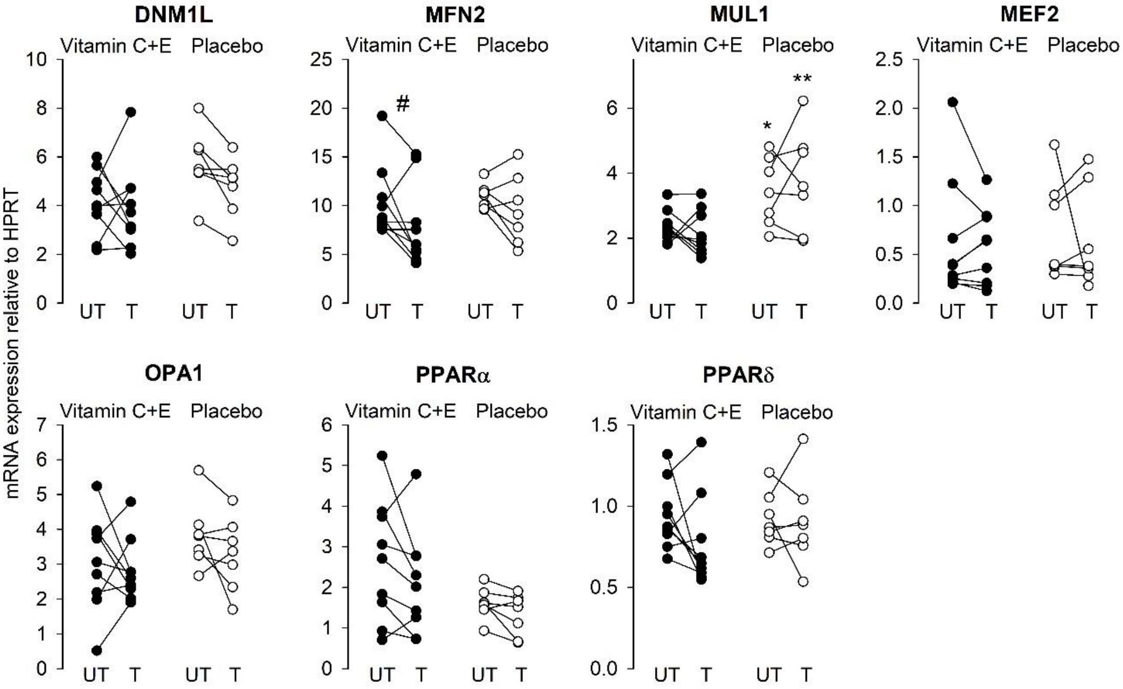

Figure S6. Gene expression of mitochondria-related proteins (other than RyR1) in the untrained state (UT, i.e. before the first SIT session) and the trained state (T; i.e. before the last SIT session). Data from vitamin-treated (●) and placebo-treated (○) individuals. mRNA expressions presented as $2^{-\Delta C_t}$, where $\Delta C_t = C_t[\text{Target}] - C_t[\text{Housekeeping}]$. Two-way repeated measures ANOVA for each gene revealed a significantly negative training effect of MFN2 in the vitamin group (# $P < 0.05$) and higher mRNA expression of MUL1 in the placebo than in the vitamin group both in the untrained (*, $P < 0.05$) and trained (**, $P < 0.01$) states; no other differences were detected between the two groups or between the untrained and trained states within each group.
